# High rates of albuminuria but not of low eGFR in Urban Indigenous Australians: the DRUID Study

**DOI:** 10.1186/1471-2458-11-346

**Published:** 2011-05-19

**Authors:** Louise J Maple-Brown, Joan Cunningham, Allison M Hodge, Tarun Weeramanthri, Terry Dunbar, Paul D Lawton, Paul Z Zimmet, Steve J Chadban, Kevan R Polkinghorne, Jonathan E Shaw, Kerin O'Dea

**Affiliations:** 1 Menzies School of Health Research, Charles Darwin University, Darwin, Australia; 2 Division of Medicine, Royal Darwin Hospital, Darwin, NT, Australia; 3 Cancer Epidemiology Centre, Cancer Council Victoria, Melbourne, Australia; 4 Department of Health, Government of Western Australia, Perth, Australia; 5 Charles Darwin University, Darwin, Australia; 6 Baker IDI Heart and Diabetes Institute, Melbourne, Australia; 7 Royal Prince Alfred Hospital and University of Sydney, NSW, Australia; 8 Department of Nephrology, Monash Medical Centre and Department of Medicine, Monash University, Melbourne, Australia; 9 Sansom Institute for Health Research, UniSA, Adelaide, Australia

## Abstract

**Background:**

Indigenous Australians have an incidence of end stage kidney disease 8-10 times higher than non-Indigenous Australians. The majority of research studies concerning Indigenous Australians have been performed in rural or remote regions, whilst the majority of Indigenous Australians actually live in urban settings. We studied prevalence and factors associated with markers of kidney disease in an urban Indigenous Australian cohort, and compared results with those for the general Australian population.

**Methods:**

860 Indigenous adult participants of the Darwin Region Urban Indigenous Diabetes (DRUID) Study were assessed for albuminuria (urine albumin-creatinine ratio≥2.5 mg/mmol males, ≥3.5 mg/mmol females) and low eGFR (estimated glomular filtration rate < 60 mls/min/1.73 m^2^). Associations between risk factors and kidney disease markers were explored. Comparison was made with the AusDiab cohort (n = 8,936 aged 25-64 years), representative of the general Australian adult population.

**Results:**

A high prevalence of albuminuria (14.8%) was found in DRUID, whilst prevalence of low eGFR was 2.4%. Older age, higher HbA1c, hypertension, higher C-reactive protein and current smoking were independently associated with albuminuria on multiple regression. Low eGFR was independently associated with older age, hypertension, albuminuria and higher triglycerides. Compared to AusDiab participants, DRUID participants had a 3-fold higher adjusted risk of albuminuria but not of low eGFR.

**Conclusions:**

Given the significant excess of ESKD observed in Indigenous versus non-Indigenous Australians, these findings could suggest either: albuminuria may be a better prognostic marker of kidney disease than low eGFR; that eGFR equations may be inaccurate in the Indigenous population; a less marked differential between Indigenous and non-Indigenous Australians for ESKD rates in urban compared to remote regions; or that differences in the pathophysiology of chronic kidney disease exist between Indigenous and non-Indigenous populations.

## Background

Indigenous Australians have an incidence of end stage kidney disease (ESKD) 8-10 times higher than non-Indigenous Australians and life expectancy 15-20 years shorter [1-3]. Whilst ESKD rates are very high amongst Indigenous Australians overall, they vary widely across Australia, with a 20-30 fold gradient reported, and a strong link to socioeconomic status [4]. Type 2 diabetes is the leading cause of ESKD in Indigenous Australians: 61% of new ESKD in Indigenous Australians was attributed to diabetes in 2005 compared to 28% in non-Indigenous Australians [5].

Fewer data are available about chronic kidney disease (CKD) amongst Indigenous Australians. Torres Strait data show that 40% of people on a local diabetes register have CKD (estimated glomerular filtration rate, eGFR, <60 mls/min/1.73 m^2^) [6]. Albuminuria rates are very high amongst Indigenous Australians [5,7-9] and are associated with components of the metabolic syndrome [7,8], predictive of kidney failure, all-cause mortality [10], and incident coronary heart disease [11].

Cigarette smoking has been associated with CKD progression in diabetes [12], and smoking rates among Indigenous Australians range from 50-70% [7,9,13].

Most studies of Indigenous Australians concern those living in rural and remote regions; less is known about patterns of kidney damage in urban areas, where the majority of Indigenous Australians live [14]. The Darwin Region Urban Indigenous Diabetes (DRUID) Study was designed to address this knowledge gap. The aims of this paper are to: (i) describe the prevalence of markers of kidney disease (albuminuria, low eGFR); (ii) determine what demographic and biomedical factors are associated with these markers; and (iii) compare risk of albuminuria and low eGFR in DRUID with that of a representative cohort of the general population from the Australian Diabetes Obesity and Lifestyle (AusDiab) study.

## Methods

### Participants

The DRUID Study was a cross-sectional study of approximately 1,000 urban Indigenous people from Darwin, Australia, undertaken from September 2003 to March 2005. The population, methods and response rates have been previously described [15]. DRUID participants met the following eligibility criteria: identified as Aboriginal and/or Torres Strait Islander; aged≥15 years; had resided within a specified geographical region around Darwin for at least six months; and living in a private dwelling. All participants underwent a 75 gm oral glucose tolerance test (OGTT) unless pregnant or on medications for diabetes. The DRUID study was a volunteer cohort including approximately 14% of the estimated target population, and therefore not necessarily representative of the target population.

### Assessment of albuminuria and low eGFR

**Figure 1:**
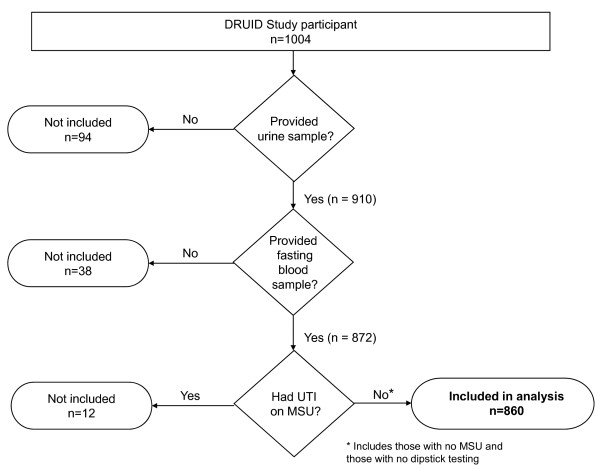
Flow diagram of included DRUID participants.

For both the albuminuria and low eGFR analyses, 860 participants provided blood and urine samples and did not have a urinary tract infection confirmed by urine microscopy and culture (Figure 1). A morning spot urine sample was performed. Dipstick testing of urine was introduced a few months after the study began [15] and was performed on all subsequent samples. Those with 1+ or greater for blood (or leucocytes or nitrites) were asked to provide a midstream urine sample (MSU). Of 197 participants requiring an MSU, 164 (83%) provided one. Participants with indeterminate results ("possible contamination, no significant growth") on MSU (n = 33) and those with no dipstick testing (n = 233) were included in the analysis.

Microalbuminuria was defined as urine ACR ≥2.5 and ≤25 mg/mmol in men and ≥3.5 and ≤25 mg/mmol in women. Macroalbuminuria was defined as ACR > 25 mg/mmol. Albuminuria was defined as ACR ≥2.5 mg/mmol in men and ≥3.5 mg/mmol in women [16].

Serum creatinine was measured by kinetic Jaffe reaction (Hitachi 917 instrument) by the Clinical Trials Laboratory, Flinders Medical Centre, prior to the introduction of creatinine assays traceable to the international reference method of Isotope Dilution Mass Spectrometry (IDMS). Low eGFR was defined as eGFR < 60 mls/min/1.73 m^2^, corresponding to CKD stage 3-5. eGFR was calculated using the "186" MDRD (modification of diet in renal disease) formula (for creatinine measurements not calibrated to IDMS) [17]. Of note, the MDRD formula has not been validated in Indigenous Australians [17,18]. However in the absence of any validated measure of GFR in this population, MDRD is currently recommended (without adjustment for "African American") [18]. Creatinine clearance was calculated using the Cockcroft-Gault formula, adjusted for body surface area (DuBois formula); neither Cockcroft-Gault nor Dubois formulae have been validated in Indigenous Australians.

### Anthropometry, blood pressure and biochemistry

Other measures were performed as described previously [15,19]. Diabetes diagnosis was based on OGTT, using the 1999 WHO diabetes classification [20]. High blood pressure was defined as systolic BP ≥ 140 mmHg or diastolic BP ≥ 90 mmHg or participant currently taking antihypertensive medications. C-reactive protein (CRP) was included as both an inflammatory marker (infection has been associated with albuminuria in Indigenous Australians [7]) and a factor associated with metabolic risk (it clusters closely with metabolic syndrome features in Indigenous Australians [21]). The metabolic syndrome was defined according to the National Cholesterol Education Panel (NCEP-ATPIII) [22].

### Comparability of DRUID and AusDiab methods

As previously described [23], the AusDiab study was a population-based survey in 1999-2000 of 11, 247 adults aged ≥25 years residing in randomly selected urban and rural areas of Australia. Methods for DRUID and AusDiab were comparable with some minor exceptions, outlined in Table 1. As with DRUID participants, AusDiab participants with confirmed urinary tract infection (n = 302) were excluded from analysis.

**Table 1:** Methodological differences between AusDiab and DRUID studies

Measure	AusDiab	DRUID
Blood Pressure	Dinamap semi-automatic oscillometric recorder	Welch Allyn Spot Vital signs monitor

HbA1c*	Boronate affinity high-performance liquid chromatography (BioRad Variant Haemoglobin Testing System, BioRad, Hercules, CA, USA)	Percentage of total haemoglobin after separation by ion-exchange chromatography on a Pharmacia Mono-S column (results traceable to DCCT method)

Serum creatinine	Modified Jaffe reaction (Olympus AU600 auto-analyser)**	Kinetic Jaffe (Hitachi 917)

eGFR	Estimated from calibrated serum creatinine values, using the "175" MDRD equation	Estimated from pre-IDMS creatinine values, using "186" MDRD equation

Urine albumin	Immunonephelometry (Beckman Array)	Immunonephelometry (Beckman array 360)

Urine creatinine	Modified Jaffe reaction (Olympus AU600 auto-analyser)	Kinetic Jaffe (Hitachi 917)

CRP	Data unavailable, therefore CRP not included in comparative model	

* Comparison of 53 samples by both HbA1c methods: mean HbA1c 5.94% (Pharmacia) vs 6.33% (BioRad), r = 0.972, p < 0.001, Bland-Altman mean difference 0.396 (95%CI 0.309-0.483), p = 0.027 (Pitman's).

**subsequent calibration to IDMS method by re-assaying a stored sub sample (n = 241)

### Statistical Analysis

Data analysis was performed using STATA v10.0 (Stata Corporation, TX, USA). Variables with distributions significantly different from normal were log transformed (natural log). Data are presented as mean (standard deviation) or geometric mean (95% confidence interval). Participant characteristics are presented by gender and comparisons were made between genders using Pearson chi-square tests (categorical variables) or independent sample t-tests (continuous variables). Univariate analysis was performed between variables outlined in Table 2 and markers of renal damage using logistic regression and unadjusted odds ratios reported. Established risk factors and variables identified in univariate analyses were then included in backward stepwise logistic regression models for albuminuria and low eGFR. Possible interactions were assessed between categorical variables of hypertension and albuminuria as well as hypertension and smoking status. Goodness of fit was assessed using likelihood-ratio tests to compare nested models; a significance level of p < 0.05 was used. Prevalence rates and 95% binomial confidence intervals of albuminuria and renal impairment are presented stratified by age group and gender for both DRUID and AusDiab participants. Pearson's chi-square test was used to compare prevalence rates of albuminuria and renal impairment between DRUID and AusDiab participants stratified by age group and gender. Data for DRUID and AusDiab participants aged 25-64 years were combined for further analysis to allow for adjustment of differences such as age distribution. Univariate associations between variables and logistic regression analysis were then performed for the combined dataset in the same manner as above.

**Table 2:** Characteristics of DRUID participants by gender.

	Male (n = 274)	Female (n = 586)
**Age **(years)	35.3 ± 14.1	37.2 ± 14.8
**Smoking Status **(%):		
**Non-smoker**:	31%	33%
**Ex-smoker**:	26%	24%
**Current smoker**:	43%	43%
**Type 2 Diabetes **(%)	15.3%	19.5%
**Height **(cm)	174.1 ± 7.1	161.9 ± 6.1^†^
**Weight **(kg)	84.0 ± 19.8	75.1 ± 20.0^†^
**BMI **(kg/m^2^)	27.6 ± 5.8	28.6 ± 7.5**
**Waist **(cm)	96.7 ± 15.3	93.6 ± 17.1**
**WHR**	0.95 ± 0.08	0.88 ± 0.10^†^
**SBP **(mmHg)	121.4 ± 15.4	114.8 ± 16.4^†^
**DBP **(mmHg)	75.2 ± 11.1	72.9 ± 9.6**
**Total chol **(mmol/L)	5.2 ± 1.3	4.9 ± 1.0^†^
**HDL chol **(mmol/L)	1.06 ± 0.30	1.19 ± 0.34^†^
**LDL chol **(mmol/L)	3.15 ± 1.01	3.02 ± 0.84
**Triglycerides **(mmol/L)*	1.77 (1.64-1.92)	1.37 (1.31-1.43) ^†^
**HbA1c **(%)*	5.58 (5.45-5.71)	5.58 (5.50-5.67)
**CRP **(mg/L)*	2.36 (2.05-2.72)	3.63 (3.28-4.10) ^†^
**Urine ACR**(mg/mmol)*	0.73 (0.60-0.88)	0.95 (0.85-1.07)**
**eGFR **(ml/min/1.73 m^2^)*	93 (91-96)	95 (93-97)

Results are mean ± standard deviation unless stated otherwise

BMI, body mass index; WHR, waist-hip ratio; SBP, systolic blood pressure; DBP, diastolic blood pressure; chol, cholesterol; CRP, c-reactive protein.

Number of participants with missing data: height - 22; weight - 27; BMI - 28; waist - 41; WHR - 42; LDL cholesterol - 35. *Geometric mean (95% confidence interval)

**p < 0.05 between genders

†p < 0.001 between genders

### Ethical Approval

Ethics approval was given by the combined Human Research Ethics Committee of Northern Territory Department of Health and Community Services and Menzies School of Health Research, Darwin. This included review by both the Aboriginal Sub-Committee and the main committee.

## Results

Characteristics of DRUID participants are outlined in Table 2. Albuminuria was present in 14.8% (n = 127) of participants: microalbuminuria 10.5% (n = 90) and macroalbuminuria 4.3% (n = 37). There was no significant difference in albuminuria between genders.

Albuminuria prevalence was lowest for participants with neither diabetes nor metabolic syndrome (8.0%) and rose steadily: metabolic syndrome (23.3%, p < 0.001 vs those without metabolic syndrome), diabetes only (27.9%), both diabetes and metabolic syndrome (33.6%). This gradient remained after adjustment for age and gender (data not shown).

Low eGFR was present in 2.4% (n = 21), with no significant difference between genders. A similar prevalence of renal impairment (2.0%) was observed using the Cockcroft-Gault formula adjusted for body surface area.

Significant univariate associations with albuminuria (unadjusted odds ratio and 95% CI) in DRUID participants were: age, 1.04 (1.03-1.06); waist circumference, 1.02 (1.01-1.04); waist-hip ratio, 212 (32-1406); mean systolic blood pressure, 1.05 (1.03-1.06); mean diastolic blood pressure, 1.07 (1.05-1.09); HbA1c, 1.6 (1.4-1.8); total cholesterol, 1.3 (1.1-1.5); HDL cholesterol, 0.3 (0.1-0.5); diabetes, 4.0 (2.9-6.1); current smoker, 1.5 (1.0-2.3); and the following log-transformed variables: triglycerides, 2.4 (1.8-3.3) and CRP, 1.6 (1.3-1.9). Significant univariate associations with renal impairment were: age, 1.08 (1.05-1.11); waist circumference, 1.03 (1.00-1.05); waist-hip ratio, 1243 (33-47468); mean systolic blood pressure, 1.05 (1.03-1.07); mean diastolic blood pressure, 1.06 (1.02-1.10); HbA1c, 1.5 91.2-1.8); HDL cholesterol, 0.1 (0.0-0.6); albuminuria, 8.9 (3.7-21.7); diabetes, 5.8 (2.5-13.5); and the following log transformed variables: triglycerides, 4.0 (2.4-6.8); CRP, 1.9 (1.3-2.7).

On multiple regression analysis (Table 3), only older age, higher HbA1c, hypertension, current smoking and higher CRP were significantly associated with albuminuria. Older age, hypertension, albuminuria and higher triglycerides were significantly associated with low eGFR, and there was a significant negative interaction (p = 0.015) between hypertension and albuminuria. Hypertension was an important risk factor for low eGFR only in participants without albuminuria; those with albuminuria had high risk of low eGFR regardless of whether hypertension was present.

**Table 3:** Final model of independent associations with albuminuria (ACR≥2.5 mg/mmol in men, ≥3.5 mg/mmol in women) and renal impairment (eGFR < 60 ml/min/1.73 m2) in DRUID participants (n = 860)

	Albuminuria OR† (95% CI)	Renal Impairment OR† (95% CI)
**Age (years)**	1.01 (1.00-1.03)	1.05 (1.01-1.10)
**HbA1c (%)**	1.33 (1.15-1.54)	-
**High BP**	2.62 (1.54-4.47)	-
Albuminuria with high BP**	-	12.94 (2.33-71.7)
Albuminuria without high BP**	-	14.8 (2.75-80.0)
High BP without albuminuria**	-	8.24 (1.54-44.1)
No albuminuria, no high BP**	-	1.0
Triglycerides (mmol/L) *		3.03 (1.52-6.04)
CRP (mg/L)*	1.22 (1.01-1.49)	
**Non-smoker**	-	
**Ex-smoker**	0.88 (0.47-1.64)	
**Current smoker**	1.79 (1.06-3.01)	

*log transformed

**†**Adjusted for all variables for which an estimate is shown

**n = 54 for albuminuria with high BP, 73 for albuminuria without high BP, 107 for high BP without albuminuria and 626 without albuminuria without high BP.

A comparison between DRUID and AusDiab participants for rates of markers of kidney damage by age group is presented in Table 4; excess albuminuria is seen in DRUID across all age groups. Table 5 presents results of multiple regression analysis for the two studies. After adjusting for other factors, being a DRUID participant was associated with a 3.2-fold greater risk of albuminuria but no significant increase in risk of low eGFR. The AusDiab study included <2% Indigenous participants (n = 116), and their inclusion or exclusion made no impact on DRUID-AusDiab comparisons (data not shown).

**Table 4:** Prevalence (% and 95%CI) of albuminuria and renal impairment by age group and gender in DRUID and AusDiab participants

	DRUID		AusDiab	
	
	M	F	M	F
Albuminuria*				
15-24 years	5.3 (1-13)	5.8 (3-11)	-	-
25-34 years	3.6 (1-12)	12.4 (7-19)	2.2 (1-4)	3.1 (2-5)^†^
35-44 years	23.9 (14-36)	11.9 (7-18)	3.6 (3-5) ^†^	3.3 (2-4) ^†^
45-54 years	22.9 (12-37)	25.2 (17-35)	4.6 (4-6) ^†^	5.0 (4-6) ^†^
55-64 years	33.3 (15-57)	21.6 (11-35)	10.0 (8-12)**	5.4 (4-7) ^†^
65-74 years	33.3 (4-77)	28.0 (12-49)	16.5 (14-19)	9.3 (8-12)**

**Renal**				
Impairment*				
15-24 years	0	0	-	-
25-34 years	0	0.8 (0-4)	0.2 (0-1)	0.6 (0-1)
35-44 years	3.0 (0-10)	0.7 (0-4)	0.7 (0-1)	1.3 (1-2)
45-54 years	6.3 (0-17)	5.6 (2-12)	2.8 (2-4)	5.4 (4-7)
55-64 years	9.5 (1-30)	7.8 (2-19)	4.7 (3-6)	9.6 (8-11)
65-74 years	16.7 (0-64)	4.0 (0-20)	18.0 (15-21)	27.3 (24-31)**

*DRUID n = 76,139; 56,129; 67,135; 48,107; 21,51; 6,25. AusDiab n = 585,775; 1086,1417; 1337,1488; 912,1027; 723,772. (for men, women respectively for each age group in ascending order)

**p < 0.05, ^†^p < 0.001: for AusDiab compared to DRUID participants of the same gender for each age group

**Table 5:** Final logistic regression models for renal impairment (eGFR < 60 ml/min/1.73 m2) and albuminuria (ACR≥2.5 mg/mmol in men, ≥3.5 mg/mmol in women): AusDiab and DRUID participants (aged 25-64 years), showing odds ratios for the variable "Druid vs AusDiab participant" for each outcome.

	Druid vs AusDiab participant		Other variables included in final model**
	**Unadjusted OR (95% CI) **^**§**^	Adjusted OR* (95% CI)	
Renal impairment (n = 8,936)	0.91 (0.57-1.46)	0.77 (0.44-1.36)	Age, gender, systolic BP, ACR, smoking status, triglycerides, BMI≥25
Albuminuria (n = 8,918)	4.24 (3.36-5.34)	3.17 (2.39-4.19)	Age, gender, high BP, waist circumference, triglycerides, smoking status

OR, odds ratio; BP; blood pressure; ACR, albumin-creatinine ratio.

Hosmer-Lemeshow test of goodness of fit: renal impairment p = 0.708; albuminuria p = 0.03.

*adjusted for other variables in final model (as listed for each outcome in final column)

**The following variables were considered for inclusion in the final model: age (10 year age group), gender, weight, BMI, waist, waist-hip ratio, BMI≥25 kg/m^2^, current smoker, high blood pressure, HbA1c, total cholesterol, triglycerides, urine ACR, and the interaction of high blood pressure and ACR. Variables with p < 0.05 were included in the final model.

^**§**^Unadjusted OR includes only those participants who had complete data for variables listed in the final model

## Discussion

Consistent with previous reports from remote Indigenous communities [7-9], we observed high rates of albuminuria (14.1%) in this urban Indigenous cohort. An unexpected finding in this study was the very low rate of low eGFR (2.4%), despite high rates of albuminuria. Compared with the general Australian population (AusDiab) and after adjustment for other risk factors, DRUID participants displayed a three-fold greater risk of albuminuria but not of low eGFR.

The relatively low rate of low eGFR is seemingly at odds with the higher rates of incident ESKD in Indigenous versus non-Indigenous Australians [1,2], but is consistent with previous observations from two remote communities in northern Australia: 53.1% albuminuria and 6.8% renal impairment (assessed by Cockcroft-Gault formula) in one community [10,24], and 44% albuminuria and 12% renal impairment (MDRD < 60 ml/min/1.73 m^2^) in another community [25]. Thus in both DRUID and two remote Indigenous studies, the proportion of participants with albuminuria was several-fold higher than the proportion with low eGFR, whereas the converse was seen in AusDiab. That the ratio of albuminuria to low eGFR is greater in Indigenous than non-Indigenous Australians is also consistent with findings of the NEFRON Study (Australian primary care) [26] and a recent report comparing First Nations People to other residents of Saskatchewan, Canada [27].

This discrepancy may reflect the different profiles of the Indigenous and general Australian groups with respect to age, disorders associated with insulin resistance, rates of diabetes or obesity-related hyperfiltration, origins and pathophysiology of kidney disease and speed of progression through CKD stages [25]. However it also raises questions regarding the appropriateness of the MDRD formula (or Cockcroft-Gault formula +/-adjustment for body surface area) to estimate GFR in this population. Creatinine-based formulae have several limitations, including lack of validation in ethnic populations apart from Caucasians and African Americans; significant differences in eGFR and reference GFR have been reported in a Chinese population (eGFR overestimated reference GFR when GFR < 30 mls/min/1.73 m^2^)[28]. Differences in body build and body composition between Indigenous and non-Indigenous Australians suggest that eGFR may under-estimate risk for Indigenous Australians [29]. It is possible that the relatively low rate of reduced eGFR in DRUID is attributable to sampling bias; however, this is inconsistent with our findings that rates of albuminuria are raised as expected.

As noted above, the prevalences of markers of kidney disease are generally lower than those previously reported in remote Indigenous communities (of similar age profile, ACR≥3.4): 14% albuminuria in DRUID compared to 28% in the Torres Strait and Northern Peninsula area [9], 38% in Central Australia [8], and 53% in the Top End [10]. These observations are consistent with the reported rates of ESKD, higher in remote than urban regions, associated with greater socio-economic disadvantage in remote communities [30]. Higher rates of diabetes in remote communities may also impact the rates of albuminuria across different Indigenous cohorts. Given this apparent urban-remote gradient, it is possible that rates of ESKD in the Darwin urban region are not disproportionately higher than non-Indigenous Australia and that much of the adverse prognosis associated with albuminuria is displayed as excess cardiovascular rather than renal risk.

The three-fold greater risk of albuminuria in DRUID participants is consistent with previous reports comparing Aboriginal and European Australians [31,32]. Predictors of albuminuria and of low eGFR in DRUID were similar to those in AusDiab [16,33], with the addition of CRP for albuminuria and triglycerides for low eGFR. High CRP levels have been reported in other studies of Indigenous Australians and are strongly related to central fat, particularly in women [34]. Measures of glycaemia or metabolic syndrome were independent predictors of albuminuria and low eGFR in DRUID participants, highlighting the importance of disorders associated with insulin resistance, and that albuminuria is a likely early manifestation of these disorders, as previously suggested by Hoy et al. [35]. Albuminuria's contribution to low eGFR in DRUID is consistent with Hoy et al.'s report of an inverse cross-sectional relationship between urine ACR and rate of loss of GFR and of baseline ACR as the most powerful predictor of longitudinal loss of renal function in a remote Northern Territory Indigenous community [24]. Indeed, albuminuria appears to be a superior predictor of accelerated GFR loss in European populations [36] and is consistent with higher ESKD rates in Indigenous versus non-Indigenous Australians. It is possible that higher rates of albuminuria in DRUID may contribute to accelerated loss of GFR in this group; this rapid GFR loss may contribute to difficulty capturing such individuals in a cross-sectional study.

### Limitations

Our study has several limitations: the DRUID study is cross-sectional and composed of volunteers (two-thirds female). The low eGFR as reported in this manuscript may not be an accurate marker of CKD: GFR was estimated from a serum creatinine assay which was performed prior to the IDMS tractable standard, was measured only once using a different assay to that utilised in AusDiab. The MDRD study equation used to estimate the GFR has not been validated in Indigenous Australians. A limitation of defining "low eGFR" is that those with pathologically high eGFR (hyperfiltration) are not in that group; differences in the rates of obesity or diabetes-related hyperfiltration between DRUID and AusDiab may have contributed to the discrepancy in our findings and warrant further study. The high rate of albuminuria but not of low eGFR may be due to the high rates of vascular and metabolic risk factors in the volunteer DRUID cohort; although factors such as raised triglycerides, central obesity and hypertension were included in the models, confounding by unmeasured factors is possible. Despite such limitations, these data represent the best currently available data for an urban Indigenous population in Australia. This is particularly important given that 73% of the total Indigenous population of Australia live in urban centres [14].

## Conclusions

We have reported high rates of albuminuria but not of low eGFR in this urban Indigenous Australian cohort. Given the high rates of ESKD observed in Indigenous versus non-Indigenous Australians, possible explanations of our findings include: (i) albuminuria may be a better predictor of ESKD in urban indigenous populations than low eGFR, as is becoming evident in other populations; (ii) shortfalls exist in the current equations when used to estimate GFR in Indigenous populations; (iii) a less marked differential between Indigenous and non-Indigenous Australians for rates of ESKD in urban compared to remote regions; or (iv) differences in CKD pathophysiology between Indigenous and non-Indigenous Australians. There is an urgent need for future work (including longitudinal studies and eGFR validation study [37]) to determine which or which combinations of these potential mechanisms explain the high rates of albuminuria but not of low eGFR in a population at high risk of ESKD.

## Competing interests

The authors declare that they have no competing interests.

## Authors' contributions

LMB planned and performed analysis, drafted the manuscript. JC and KOD were DRUID study investigators, contributed to the analytical design and intellectual input to manuscript. AH, KP contributed to the analytical design and manuscript preparation. TW, TD, PL, PZ, SC and JS were DRUID study investigators and contributed to manuscript preparation. All authors approved the final manuscript.

## Pre-publication history

The pre-publication history for this paper can be accessed here:

http://www.biomedcentral.com/1471-2458/11/346/prepub
